# Strategies Regarding High-Temperature Applications w.r.t Strength, Toughness, and Fatigue Life for SA508 Alloy

**DOI:** 10.3390/ma14081953

**Published:** 2021-04-13

**Authors:** Muhammad Raies Abdullah, Cai Hongneng, Fang Liang

**Affiliations:** 1 Key Laboratory for Mechanical Behavior of Materials, Xi’an Jiaotong University, Xi’an 710049, China; raieskhan@stu.xjtu.edu.cn (M.R.A.); hntsai@mail.xjtu.edu.cn (C.H.); 2 School of Mechanical & Electrical Engineering, Xiamen University Tan Kah Kee College, Zhangzhou 363105, China

**Keywords:** computational thermodynamics, Thermo-Calc, CALPHAD, database, SA508, RPV

## Abstract

In this work, the stabilities of secondary phases, including carbides, brittle phases, and inclusions, were simulated by computational thermodynamics. Calphad strategical optimization is preferable for all steel alloys regarding energy resource consumption during manufacturing and processing. The alloy composition has been changed to enhance the strength, hardenability, and longevity of a reactor pressure vessel (RPV) steel by computing the phase equilibrium calculations and predicting mechanical properties such as yield and tensile strengths hardness and martensitic and bainitic volume fractions. The stabilities of the pro-eutectoid carbides (cementite), inclusions, and brittle phases in SA508 steel are critical to the toughness and fatigue life related to the crack initiation and expansion of this steel. Overall, the simulations presented in this paper explain the mechanisms that can affect the fatigue resistance and toughness of steel and offer a possible solution to controlling these properties at elevated temperatures by optimizing the steel composition and heat treatment process parameters.

## 1. Introduction

### 1.1. Background

Reactor pressure vessel (RPV) materials are necessary to achieve mechanical properties that can withstand the high pressure inside reactors, one of the significant factors in deciding the reactor’s durability and safety margin. As a result, the demand for materials with higher strength, hardness, and low-fatigue characteristics increases nuclear power plant generation capability and operating life. One of the most critical reactor problems for certain RPV materials is the resistance to irradiation embrittlement, which causes significant toughness loss [1]. In the ductile-brittle transition zone, ferritic steels such as bainitic and pearlitic steels showed a significant decrease in fracture toughness as the temperature decreased [2]. ASME SA508 has been in use for decades as it is an acceptable solution balancing strength, resilience, and costs, and it currently satisfies the service requirements [3,4,5]. SA508 tempered a bainite microstructure consisting of ferrite and carbide and ferritic laths in low-alloy steel [6]. In developing new types of steel, the economic viability should also be in mind, and we should minimize the alloy additions, which significantly increase costs. This steel class owns a higher density, strength, high stiffness, fracture toughness, and excellent corrosion resistance than the light metals (Al, Mg, Ti) alloys [3]. These steels have complex, multi-phase austenite (FCC), martensite, ferrite (BCC), and finely scattered carbide microstructures. Phase fractions play a critical role in the success of exceptional creep and fatigue properties, and they have been extensively studied [7,8,9].

High temperatures, pressures, corrosive environments, and neutron irradiation are all conditions that RPVs must work. These requirements cause the use of structural materials with exceptional properties. Most pressurized water reactors (PWR) operate between 270 and 330 °C at a significant pressure of 15–23 MPa under normal operating conditions. Any new material must show improved resilience, hardness, and strength, as the RPV is the essential life-limiting portion. We chose three different alloy compositions in the present work, as shown in Table 1, based on the alloy chemistry reported for the 508 steel class [10], keeping in mind the physical metallurgical aspects. The study aims to identify the optimum number of alloying elements (Mn, C, Si), which maintain the perfect combination of hardness, strength, toughness, and fatigue life. The mechanical properties evaluated by JmatPro (version 7.0) (Sente Software, Guildford, United Kingdom) software are reported [11].

### 1.2. Computational Specifications

The alloy compositions (Table 1) shows the chemical composition of SA508 with different concentrations of Mn, Si, and C. As for the problem of increasing the Ac_3_ (ferrite(α) to austenite (γ) transition temperature), and Ac_4_ (austenite (γ) to ferrite (δ) transition temperature) critical temperatures for setting casting and forging parameters [3]. The issue occurs when undesirable phases become more stable during heat treatment, such as δ-BCC and brittle phases, reducing its toughness and longevity in high and low-temperature applications. Carbide-forming elements alloyed in steels can result in different kinds of reinforcements, such as MC, M_3_C, or M_6_C carbides, giving rise to a wide range of properties. Typically, MC carbides exhibit the best properties in terms of hardness, while M_3_C carbides, such as cementite, are weaker, and M_6_C carbides show intermediate properties [4]. It is then possible to tune steel properties by combining different carbides, depending on the final application.

The Calphad approach marks a shift in alloy design, which currently aims to realize new properties through chemical composition changes. The design pattern of alloy chemistry focuses on the behavioral study of phase fractions and their consequences for structural and mechanical properties. For calculations related to SA508 analysis, 1100 K is austenitization temperature and austenite grain size has been supposed to be 10–15 microns. The benefits and the detrimental consequences of each alloy factor must be taken into consideration when designing alloys. It is common knowledge that austenite stabilizers like C and Mn increase the stability of γ, reduce martensite start (Ms) temperatures while Si increases the temperature of austenitization, as shown in Figure S3 of the Supplementary Materials. The morphology and number of secondary phases depend on the amount and combination of alloy components, mainly carbon content. carbon preferentially partitions into austenite at intercritical annealing temperatures due to its poor solubility in ferrite. Due to the higher cost, Ni content should be kept to a minimum. Silicon is a ferrite modulator that improves the ferrite matrix by solid solution strengthening [5]. Besides, it promotes carbon partitioning in retained austenite by suppressing carbides [6], thereby enhancing its stability. However, the addition of Si above 0.5 wt.% causes problems in galvanization, welding, and casting phenomenons [7]. Cr and Si are known to regulate cementite dissolution rates and, as a result, improve fatigue properties by forming fine carbide particles [8]. The susceptibility of low-partition components in the alloy, particularly sulfur, phosphorous impurities, and niobium alloyed elements, deteriorates as the temperature range increases due to segregation [9]. However, Nb is a strong carbide former such that a small amount of Nb increases strength through the precipitation of MC-type carbides [10]. Based on the above considerations, we restrict the compositional space for C up to 0.15–0.45 wt.%, Mn up to 0.45–1.05 wt.%, and Si up to 0.15–0.45 wt.%. The elemental alloying properties, such as atomic weight, partition coefficient, and the chemical potential of the secondary phases, also affect the solidification segregation and formation of the secondary phases.

The two leading Calphad-based software programs have been used to propose the alloying composition for different SA508 steel samples. Jmat-pro is used to calculate the hardness and strength of alloy concerning different compositions of Mn, Si, and C by comparing their hardness, yield, and tensile strengths [12]. Thermocalc (2019a) (Royal Institute of Technology (KTH), Stockholm, Sweden) calculates the phase properties at different temperatures with the TCFE9 database package. TCFE9 is a thermodynamic database for different kinds of steel and Fe-based alloys, including stainless steel, tool steels, corrosion-resistant steels, low-density steels, HSLA steels, and also cemented carbides. This database is valid for the simulation of the solidification process, the stability of matrix phases (γ and α), precipitation of secondary phases such as sulfides, carbides, nitrides, and intermetallic phases as Sigma and Laves phases [13]. JmatPro has also developed in Calphad databases, which can be usefully employed for calculating various mechanical properties, as well as thermodynamic properties of different kinds of alloy along with steel alloys [14]. This software is utilized to understand the overall trends of mechanical properties such as hardness, thermal expansions, rupture life, time-temperature-transformations (TTT), and continuous-cooling-transformations (CCT) diagrams of multicomponent alloys [15]. The range of composition of carbon in 508 Gr 1 was around 0.35 wt.%. In contrast, Si was about 0.15–0.45 wt.%, while S and P were in minimal concentration. The composition of Mn is around 0.45–0.05 wt.% as shown in Table 1.

## 2. Secondary Phases (G-Phase, Cementite, Carbides, and Inclusions) Effects on Mechanical Properties of SA508 Steel

In the initial microstructure, finely distributed M_23_C_6_ carbide particles in the matrix strengthen the steels but the M_23_C_6_ carbide is also undesirable because of its intergranular corrosion and decrease in ductility and toughness [16]. Conversely, the presence of M_23_C_6_ on the grain boundaries can make sliding more difficult for grain boundaries, therefore, improving creep ductility [16]. Usually, grain boundaries enable the alloying elements to diffuse by coarsening and nucleation of the M_23_C_6_ carbides. Stable precipitates of M_23_C_6_ block the grain boundaries, so under the effect of creep stress, grain boundary energy will be higher, which forces vacancies in the matrix to be stored in that area where stress concentration is produced. The gap between the grain boundaries and carbides grows, which leads to the coming apart of carbides from the matrix, so creep rupture starts in the separation process, which leads to fracture [17]. The high melting point of MC-type carbides (NbC, VC, MoC) makes them perfect for high-temperature structural applications, as they maintain a large portion of their strength and hardness while gaining ductility. Their negative aspect is their brittleness at room temperature, which is due to the ease with which cracks can evolve and propagate in these low-toughness phases. The growth of MC phases increases the susceptibility to cracking, corresponding to solidus temperature rise [18]. Because of their high thermodynamical stability and low solubility in liquid iron, the microstructure’s modification by thermal treatment following carbide formation can be very complicated and can require very high temperatures. In steels, cementite, a metallic carbide with the chemical formula Fe_3_C, can improve the mechanical properties over pure iron. If intermetallics can positively influence the strength, they also often reduce the ductility and the fracture toughness of the phase in which they are embedded. Precipitation of intermetallic phases from γ usually relates to detrimental consequences like matrix impoverishment in alloying elements such as chromium, molybdenum, and niobium verdict decrease ductility and toughness, a phenomenon known as embrittlement. For practical combinations of austenite grain size and cooling intensity, allotriomorphic ferrite, which is originally thought to contribute to a degradation in hardness, is contained in the microstructure. During the process of γ → δ transformation, δ-ferrite grains are continuously grown through elemental diffusion, so the volume fraction increased, and they become coarser, which makes their presence detrimental for ductility and toughness [19]. In steels, MnS inclusions help to improve machinability while also restricting grain development. Meanwhile, the morphology of these sulfide inclusions has a significant impact on a variety of steel properties. Manganese sulfide has also harmed steel alloy mechanical properties and corrosion parameters [20]. MnS inclusions are a stress raiser with soft nature, low-shear strength, and excellent ductility such that the cutting resistance of steels and the tool wear rate are reduced. The G-phase as an intermetallic phase (the chemical composition following the ratio Ni:Mn:Si = 16:6:7 is denoted as Ni16Mn6Si7) at the α/α interface, along with spinodal decomposition. The G-phase improves strength and brittleness by interacting with mobile dislocation and decreases toughness and ductility [9].

## 3. Results and Discussions

### 3.1. Phase Transformations

**Figure 1:**
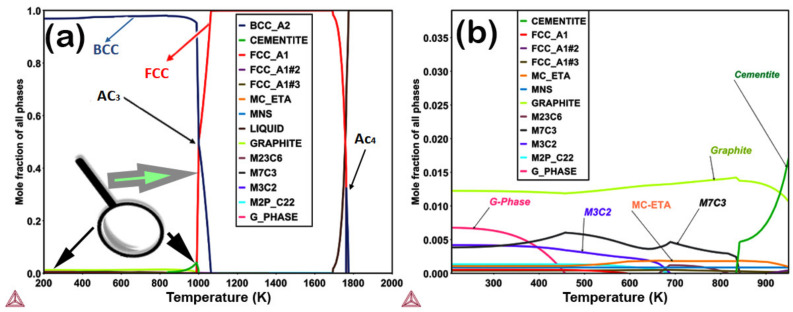
(**a**) Stable phase diagram of SA508 w.r.t to the temperature at 200–2000 K; (**b**) 200–1000 K range temperature area of stable phases (Zoom area).

Thermocalc was used to calculate the phase fractions present in the SA508 steel grade, i.e., austenite γ-FCC(FCC_A1), ferrite α-BCC(BCC_A2), cementite, carbides (M_23_C_6_, MC-ETA, M_3_C_2_, M_7_C_3_), MnS, G-phase, M2P_C22, graphite, and liquid by changing the temperature in SA508 steel as shown in Figure 1a. α-BCC is stable at room temperature and capable of containing up to only 0.008 wt.% carbon. In SA508 composition, some influential α-BCC formers are, e.g., Cr, Si, and some α-Fe stabilizers are, e.g., Si, and P [21]. As alloying elements deal with cementite, they either serve as segregating elements with a comparatively strong solubility in cementite (e.g., Mn, Cr, and Mo) or as anti-segregating elements with a minimal cementite solubility (e.g., Si) [22]. The critical temperatures are Ac_3_ (transformation of α → γ phase), and Ac_4_ (transformation of γ → δ phase) during phase transformations, as shown in Figure 1a for SA508 steel. At Ac_4_ temperature, γ transforms into δ-iron due to the higher solubility of phosphorus and sulfur in the δ-phase than that in the γ phase, prominent to the enhancement of ductility by stifling of grain boundary segregation [23]. Alternatively, when the amount of δ-ferrite is as high as 7–10%, ductility deteriorates dramatically due to the strain concentration at the interface induced by the difference in deformation resistance between the and the δ and the γ phase. Therefore, adjusting the δ-ferrite is essential for decreasing hot-working associated flaws [24]. The Ac_3_ critical temperature is an essential parameter in casting, austenitizing, and annealing phenomena to make stable phases and remove brittle phases that reduce ductility and toughness [25]. As shown in Figure S3, Ac_3_ temperature ranges between 1100–1170 K for different alloying elements.

As we considered low C concentrations according to ASME standard, our study mainly focused on γ-FCC, α-BCC, M_23_C_6_, MC-ETA, M_3_C_2_, M_7_C_3_, and G-phase, shown as highlighted in Figure 1. Figure 1 shows all phases for the RF sample of SA508 grade steel, including secondary precipitates, which have detrimental effects on the toughness and ductility of stainless steel at high-temperature applications. Increasing carbon decreases the Ac_3_ and Ms temperature (Figure S3a), but it also increases the mole fraction of δ-phase, as shown in Figure S2a. Mn decreases the Ac_3_ temperature along with decreasing Ms temperature, as shown in Figure S3a. Mn tends to decreased the δ-phase mole fraction, as shown in Figure S2b. Si is a strongly oxidizing agent at both high and low temperatures. It promotes a *δ*-ferritic structure at high temperatures like 1700–1800 K, and, at low temperatures 300–1000 K, it decreases the ferritic behavior of alloys, as shown in Figure S2c. MC-ETA, G-phase, M_7_C_3_, and M_3_C_2_ phases are stable at low temperatures, even 200 K, as shown in Figure 1b. The main objective for using CALPHAD methodology in this study was to check the alloy’s solidification behavior regarding alloying elements, i.e., Mn, Si, and C. The solidification path in SA508 alloy is L **→** L + α **→** L + α + γ **→** L + γ + MnS **→** L + γ + MnS + graphite **→** L + γ + MnS + graphite + cementite between 1500–1100 K temperatures, as shown in Figure S8.

### 3.2. Effect of Carbon

**Figure 2:**
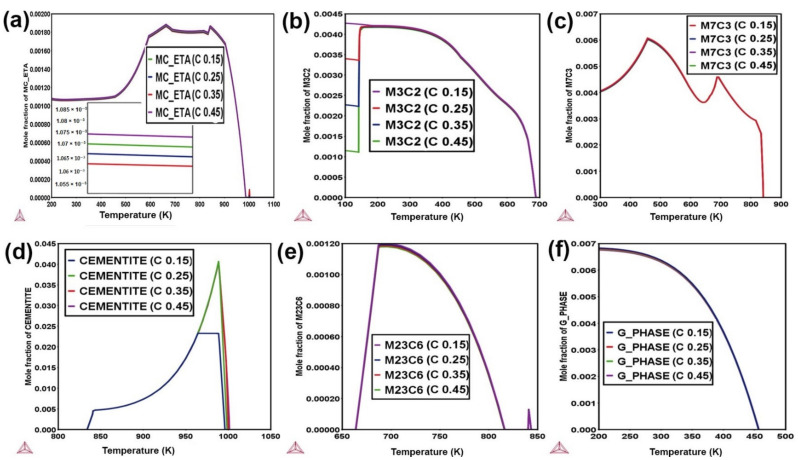
Effect of C concentration to MS1 alloys for, (**a**) MC_ETA; (**b**) M_3_C_2_; (**c**) M_7_C_3_; (**d**) cementite; (**e**) M_23_C_6_; (**f**) G-phase.

Figure 2 illustrates the phase transformations of MS1 alloys that have C concentrations of 0.15–0.45 wt.%. Large phase stabilities are observed for M_23_C_6_, M_3_C_2_, M_7_C_3_, MC, cementite, and G-phase with changing C concentration. carbon’s effects are particularly noteworthy among the numerous components. carbon content influences grain boundary mobility, which affects final grain size and phase transformation [26]. High carbon content raises the carbide fraction volume and creates a stronger and more wear-resistant steel but with reduced toughness [9]. Increasing the C content increases the stability of the MC-ETA carbide at high-temperature (1000 K), but at low temperature, it decreases the stability of MC-ETA, as shown in Figure 2a. In SA508 grade, MC carbide composition belongs to M(V, Mo, Nb)C as shown in Figure S4a. carbon increases the molar fraction of cementite (Fe_3_C) from 0.020 to 0.040-mole fraction, which has adverse effects on the ductility and toughness of steel alloys at high-temperature applications. Cementite is stable at 840–1000 K, but at lower temperatures, Cr and Mn have higher site fractions than Fe, as shown in Figure S5d. With a lower carbon content, the likelihood of the formation of cementite precipitates is reduced. These cementite inter-lath precipitates, which are known sites for fracture initiation, are reduced in size with decreased carbon content, leading to increased cleavage fracture stress. An increase in M_23_C_6_ carbide has adverse effects on ductility and toughness at higher temperatures because M_23_C_6_ has a higher coarsening rate at high temperatures, i.e., the Ostwald ripening effect [27]. As the G-phase is an intermetallic brittle phase, it has complex behavior in SA508 alloys, i.e., stable at low temperatures from 200 to 450 K but, after that, it diminishes as shown in Figure 2f.

M_23_C_6_ is found primarily in martensitic laths with high-density dislocations, causing coarsening and faster dislocation recovery, resulting in elongated subgrains and shortened creep life. Changing the carbon concentration does not affect so much the distribution of G-phase and carbides, mostly in high Cr alloys [9]. As discussed, the concentration of carbon cannot decrease drastically, as strength cannot be sacrificed.

### 3.3. Effect of Manganese

**Figure 3:**
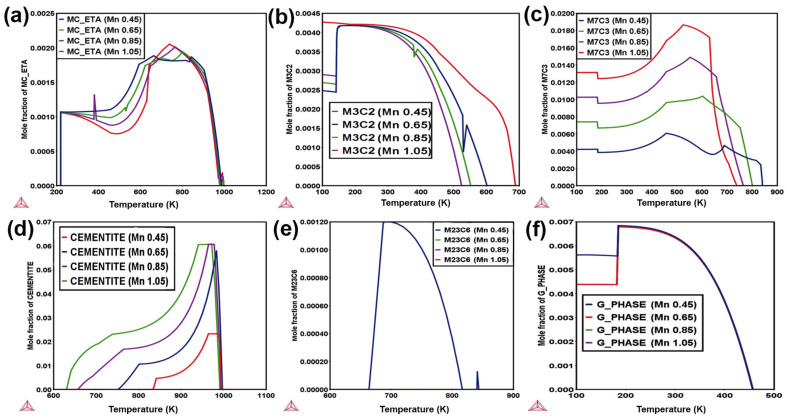
Effect of Mn concentration to MS2 alloys for, (**a**) MC_ETA; (**b**) M_3_C_2_; (**c**) M_7_C_3;_ (**d**) cementite; (**e**) M_23_C_6_; (**f**) G-phase.

Figure 3 illustrates the phase transformations of MS2 alloys that have Mn concentrations of 0.45–1.05 wt.%. Large phase stabilities are observed for MC-ETA, M_23_C_6_, M_3_C_2_, M_7_C_3_, cementite, and G-phase with changing Mn concentration. Manganese is commonly used in steel alloys to increase hot ductility. Its effect on the α/γ balance varies with temperature [28]. Mn increases the stability range of temperature (650–1000 K) and a molar fraction (0.02–0.06) of cementite, as shown in Figure 3d. Regarding M_23_C_6_ carbide changing the concentration of Mn has anomalous behavior because it just shows the stability at 0.45 wt.% as shown in Figure 3c.

Mn stabilizes the M_3_C_2_ carbide from 530 to 700 K temperature. While for M_7_C_3_, it reduced the temperature range from 850 to 750 K, but it increases the molar fraction M_7_C_3_ from 0.004 to 0.014 mole. At low temperature (200–500 K) in the composition M(Fe, Cr, Mn)_7_C_3_, Mn is at high content and then replaced by Cr and Fe, as shown in Figure S4c. Regarding site fractions of M_7_C_3_, Mn has more site fractions at low temperature until 500 K and is then replaced by Fe and Cr (Figure S5c). Regarding MC-ETA, at 500 K, it reduces the molar fraction, but at 800 K, it increases Mn content, which increases the molar fraction until 0.002-mole fraction, as shown in Figure 3a. The G-phase still has complex behavior like MS2 alloys, although at low temperature (100–200 K) Mn decreases the stability of the G-phase, later (200–460 K) it has the same molar fraction for both 0.45 and 0.65 wt.% of Mn, as shown in Figure 3f. Mn is an austenite stabilizer at low temperatures till 1000 K along with decreasing behavior of Ac_3_ temperature, as shown in Figure S2b. Mn was introduced into steels as a replacement for Ni in the international market. Researchers are interested in producing medium Mn steels as part of the 3rd generation of advanced high-strength steels because of the prospect of obtaining a high tensile strength-high ductility combination at an inexpensive rate [28].

### 3.4. Effect of Silicon

**Figure 4:**
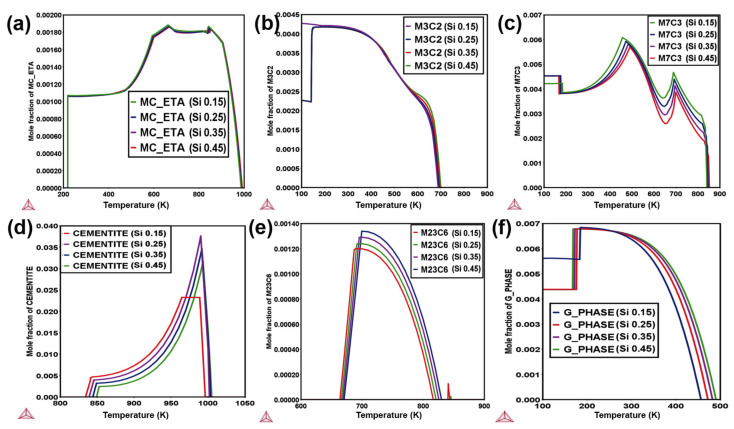
Effect of Si concentration to MS3 alloys for, (**a**) MC-ETA; (**b**) M_3_C_2_; (**c**) M_7_C_3_; (**d**) cementite; (**e**) M_23_C_6_; (**f**) G-phase.

In Figure 4, the graph shows the phase transformations of MS3 alloys that have silicon concentrations of 0.15–0.45 wt.%. Large phase stabilities are observed for MC-ETA, cementite, carbides, and G-phase with changing Si concentration. In precipitation, Si is trapped in cementite at the initial stage but rejected from cementite after tempering. Si is sometimes used to increase the stability of austenite that has been retained, as shown in Figure S6c, by delaying cementite formation and the associated consumption of carbon, as shown in Figure 4d. In silicon-containing steels, the austenite keeps carbon in a solid solution, enabling it to remain untransformed at room temperature. The solubility of Si in cementite is almost negligible, so Si’s entrapment is assumed to significantly reduce the driving force for cementite precipitation at low temperatures [6]. However, to prevent the forming of an adherent red-scale on the steel surface, the silicon concentration must be optimized [29]. It promotes a *δ*-ferritic structure at high temperatures like 1400–1500 K, but at low temperatures 600–1200 K, it increases the austenitic behavior of alloys, as shown in Figure S2c. As shown in Figure 4e, it frequently increases the molar fraction of M_23_C_6_ regarding the content of Silicon.

Si additions accelerate the Mn partitioning kinetics and decelerate the particle growth kinetics [30]. Si decreases the molar fraction of the M_7_C_3_ phase, the highest molar fraction is at 450 K, as shown in Figure 4c. MC-ETA phase is stable from 200–1000 K temperature, Si content also decreases the MC-ETA phase’s stability regarding molar fraction, but it does not affect it too much, as shown in Figure 4a. In 508 steel, the G-phase is primarily dependent on Si content, it shifts the G-phase’s stability at higher temperatures, i.e., 470–505 K temperature, as shown in Figure 4f. Si is also used as a beneficial alloying element to reduce the density of steel [31] and hinders the cementite from γ, causing the remaining γ to become enriched with carbon, which is retained in the final precipitates [32].

### 3.5. Mechanical Properties

**Figure 5:**
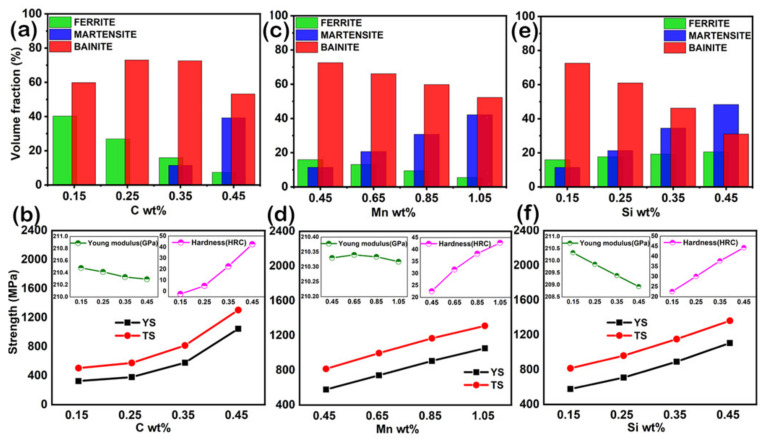
Effect of C content (**a**) Volume fraction of phases; (**b**) Tensile strength, yield strength, Young’s modulus and hardness of MS**1** alloys at room temperature, effect of Mn content; (**c**) Volume fraction of phases w.r.t Mn; (**d**) Tensile strength, Yield strength, Young modulus, and hardness of MS**2** Alloys at room temperature, effect of Si content; (**e**) Volume fraction of phases w.r.t Si; (**f**) Tensile strength, yield strength, Young modulus, and hardness of MS**3** Alloys at room temperature.

Figure 5 shows the mechanical properties of Cr, Mn, and Si in SA508, i.e., modulus, hardness, yield strength, tensile strength, and volume fraction of phases using Jmat-pro at 300 K test temperature. These SA508 steel concentrations have been optimized to find the best possible combination of strength, hardenability, and creep resistance. First, the effect on mechanical properties and phase transformations regarding carbon has been investigated, as shown in Figure 5a,b. As previously mentioned, carbon induces solid-solution strengthening, so both the YS and TS rise as the C concentration increases at a faster rate than other elements (Figure 5b). Meanwhile, C increases the hardness from 2–45 HRC, but it decreases the Young’s Modulus, which is related to its creep life, as shown in equation A1. Creep properties are also related to the alloy modulus, so that the higher the Modulus, the better creep resistance [33].

As for the RF sample of SA508 Gr.1 is a mostly bainitic structure as shown in Figure 5. The bainite microstructure material shows a much longer fatigue life from a metallurgical perspective than with ferrite/pearlite microstructure. The characteristics of crack propagation in different microstructures seem to be the leading cause of fatigue life. In a hypo-Eutectoid range (0–0.80 wt.% C), C alloying reduces the volume fraction of ferrite while raising the volume of bainite to 0.35% and reducing the volume fraction to 0.45% (Figure 5a). Nonetheless, since strength cannot be sacrificed, the C concentration cannot be significantly decreased. Regarding pearlite and austenite fractions, C also increases the molar fraction at room temperature, but those fractions are so small compared to other major phases, as shown in Figure S6a.

The following method is the exact measurement for various Mn concentrations, which has fixed the other alloy element contents as Fe-0.35C–(0.45–1.05)Mn–0.15Si-other fix elements. As shown in Figure 5d, Mn also enhances the alloy’s YS and TS as it works as a solid solution strengthening alloying element. However, the Young’s Modulus curves’ slope increases until 0.65 wt.%, it gradually decreases. Regarding hardness, it increases the hardness of alloy from ~20 to ~40 HRC. The volume fraction of bainite and ferrite is decreased from 72–52% and 15–5%, respectively, while the martensitic phase fraction increases with the addition of Mn content from 11–42% in phase fraction, as shown in Figure 5c. Mn’s addition also significantly raises the austenite volume and reduces the pearlite volume (Figure S6). The exact measurement was done for various Si concentrations, and the content of the other alloying elements was fixed as Fe-0.35C–(0.45)Mn–(0.15–0.45)Si-other fix elements. As shown in Figure 5f, Si also enhances YS and TS of the alloy from 578–1107 MPa and 816–1363 MPa, respectively. However, the slopes of the Young’s Modulus curves decrease with the increasing content of Si. Regarding hardness, it increases the hardness of alloy from ~26 to ~46 HRC. The volume fraction of martensite and ferrite increases from 11–48% and 15–20%, respectively, while the bainite phase fraction decreases from 72–30% with the addition of Si content, as shown in Figure 5e. However, the most crucial factor in optimizing the Si composition is that Si contributes to increased strength and toughness via fine carbide particle formation during the heat treatments. Si also reduced alloys creep life because it decreased the Youngs Modulus of steel grade, so proper optimization should be necessary between creep, fatigue, and toughness according to the application’s service condition. Regarding austenite and pearlite, Si also increases the volume fraction of both phases with Si in the steel grade, as shown in Figure S6.

## 4. Proposed Composition Regarding High Toughness and Fatigue Properties at 650 K

**Figure 6:**
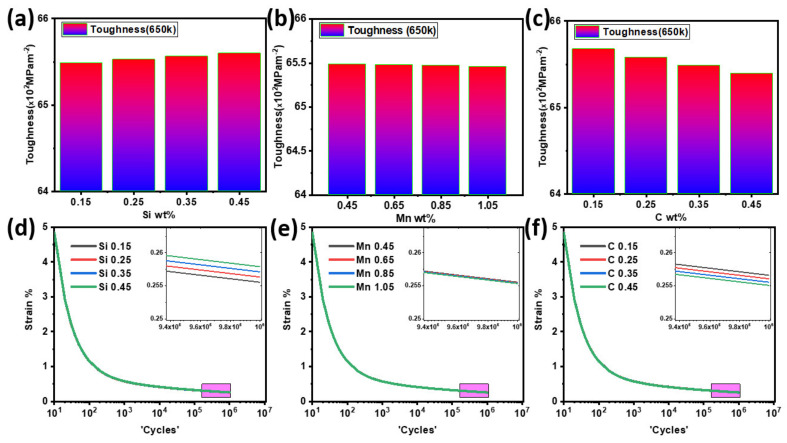
Jmat-Pro simulations for (**a**) Toughness at 650 K w.r.t Si wt.%; (**b**) Toughness at 650 K w.r.t Mn wt.%; (**c**) Toughness at 650 K w.r.t C wt.%; (**d**) Fatigue rate at 650 K w.r.t Si wt.%; (**e**) Fatigue rate at 650 K w.r.t Mn wt%; (**f**) Fatigue rate at 650 K w.r.t C wt.%.

Based on the aforementioned discussions, we suggested a new composition of the SA508 alloy, which has high toughness, optimized strength and hardness, better fatigue, and creep-rupture life for high-temperature applications up to 650 K temperature. The reason for changing the composition of alloying elements like C, Si, and Mn is to tune the mechanical properties by phase transformations of alloy, i.e., M_23_C_6_, M_7_C_3_, MC G-phase, which are our main focus to minimize or optimize according to the requirement of the application. The proposed compositions are tabulated in Table 1 along with ASME standards and reference baseline for SA508. We use a 0.1 strain rate and 650 K test temperature for stress-strain curves shown in Figure S7. Figure S7 shows that increasing Mn and C concentration decreases the stress-strain ratio, while increasing Si content increases this ratio, reflecting its effects on toughness values, as shown in Figure 6. The toughness of 508 steel is calculated by the trapezoidal rule, i.e., integrating the area under the stress-strain plot (Figure S7). The phase of ferrite is soft and ductile, while pearlite is stiff and brittle. With the carbon content, the perlite volume rises while the ferrite volume decreases. The bulk strength increases and the ductility drops as the perlite volume fraction rises. As stated above, it was found that a decrease in the C concentration would enhance the toughness of the steels, which also reduced the stability of cementite and carbides, detrimental to ductility and toughness, as shown in Figure 2. Higher carbon leads to carbide particle precipitation, which gradually aggregates near the matrix’s boundaries and causes repeated cyclic load failure, reducing fatigue at high-temperature applications. The optimal carbon content for SA508 in the investigated ranges is at about 0.15 wt.%. Mn is optimized at 0.65 wt.% for two reasons: first, after this concentration, it decreases the Young’s modulus, which reduced the creep life, and second, after 0.65 wt.%, the stability of M_23_C_6_ and G-phase is decreased as shown in Figure 3, both of which are fruitful for ductility and toughness for high-temperature applications. However, it also increases the stability of FeC_3_ and M_7_C_3,_ which causes reduced toughness.

Regarding MnS inclusions, which are detrimental LWR reactors, we use an optimum 0.65 wt.% of Mn in SA508 for RPV applications. As shown in Figure 6d, increasing Si reduces the strain rate at high cycle aggregates to decrease the fatigue rate of an alloy at 650 K. As previously mentioned, Si helps to increase fatigue intensity and hardness by shaping fine carbide particles, as reflected in Figure 6a,d. Si increases hardness and strength in the mechanical properties aspect, so a higher concentration of 0.45 wt.% of Si will be fruitful for our alloy composition. According to the fatigue model (Equation (S2)) simulation results and the above discussion, the modified composition for high temperature (650 K) applications is shown in Table 2.

Alloying elements work for two fundamental factors: (1) hardenability and (2) producing thermally stable carbides. Carbides provide resistance to (abrasive) wear on the matrix material, increasing the yield strengths. On the other hand, proeutictic carbides (cementite), coarse carbides, white matter, and inclusions (MnS) initiate fractures that are detrimental to alloy fatigue life for specifically RPV’s steel components. According to the CCT calculation shown in Figure S1a, 10 K/s cooling rates create the bainitic structure of SA508 steel. In SA508, toughness and fatigue life can be controlled by manufacturing processing according to the CCT diagram (Figure S1) to produce the clean and nano-structure bainite mix with fine plates of carbides, along with avoiding void formation interface during the forming operation and debonding then it could have high toughness and more fatigue life. As shown in Figure S3, the minimum temperature for austenitization is around 1070–1170 K for SA508 steel.

## 5. Conclusions

In this work, we propose a computational method in Calphad, based on optimizing the SA508 compositions according to their phase transformations and mechanical properties such as hardness, tensile strength, ultimate strength, toughness, and fatigue high-temperature applications.

The functions of various alloying elements in SA508 were investigated computationally based on secondary phase (MC-ETA, cementite, carbides, and G-phase) stability and different transition temperatures (Ac_3_, Ac_4_). The specific elements (C, Mn, Si) were selected in simulations as their crucial rule of affecting the mechanical properties along with the stability of major secondary phases in the SA508 alloy. These simulation results indicate that a decrease in C concentration increases the toughness and decreases the fatigue rate of the alloy, as shown in Figure 6. Increasing Mn increased the stability of cementite and carbides detrimental to toughness and the fatigue rate of alloy for specific applications of RPV, the lower concentration required for machine-ability and formability of alloy (0.65 wt.%) were selected. Since Si plays a vital role in retarding cementite formation, as seen in Figure 4, fine carbide particles with a sufficient Si concentration can improve both strength and hardness. Therefore, it is concluded that an increase in Si (0.45%) concentration is necessary to decrease the fatigue rate of alloy. Regarding the optimization of heat treatment, 1100 K temperature is selected for austenitization with a 10 K/s cooling rate to acquire the ideal mechanical properties of the bainitic structure of SA508 steel. Further optimization and experimentation are still needed for optimization according to application requirements.

## Figures and Tables

**Table 1:** Chemical compositions in SA508 Gr 1(wt.%) [11].

SA508 Gr1	C	Mn	P	S	Si	Ni	Cr	Mo	V	Nb	Fe
ASTM Standard	0.15–0.45	0.45–1.05	0.025	0.025	0.15–0.45	0.40	0.25	0.10	0.05	0.01	Bal
Reference Sample (RF)	0.35	0.45	0.025	0.025	0.15	0.40	0.25	0.10	0.05	0.01	Bal
MS1 (Modified Sample C)	0.15–0.45	0.45	0.025	0.025	0.15	0.40	0.25	0.10	0.05	0.01	Bal
MS2 (Modified Sample Mn)	0.35	0.45–1.05	0.025	0.025	0.15	0.40	0.25	0.10	0.05	0.01	Bal
MS3 (Modified Sample Si)	0.35	0.45	0.025	0.025	0.15–0.45	0.40	0.25	0.10	0.05	0.01	Bal

**Table 2:** Modified chemical compositions in SA508 Gr 1 (wt.%) along with the ASTM standard [11].

Alloy	C	Mn	P	S	Si	Ni	Cr	Mo	V	Nb	Fe
Modified Sample	0.15	0.65	0.025	0.025	0.45	0.40	0.25	0.10	0.05	0.01	Bal
ASTM Standard	0.15–0.45	0.45–1.05	0.025	0.025	0.15–0.45	0.40	0.25	0.10	0.05	0.01	Bal

## Data Availability

The data presented in this study are available on request from the corresponding author. The data are not publicly available due to privacy restrictions.

## Supplementary Materials

The following are available online at https://www.mdpi.com/article/10.3390/ma14081953/s1, Figure S1: Jmat Pro calculation for SA508 grade steel for RPV (**a**) Continuous Cooling Transformation (CCT) for SA508 (**b**) Isothermal transformation (TTT) for SA508 steel, Figure S2: Ac_3_(ferrite to Austenite transition temperature) and Ac_4_(Austinite to Ferrite transition temperature) temperature calculations (**a**) FCC and BCC for MS1 samples; (**b**) FCC and BCC for MS2 samples; (**c**) FCC and BCC for MS3 samples, Figure S3: Martensite temperature start (Ms), Ac_3_ and Ac_4_ temperatures w.r.t (**a**) MS1 (changing Carbon contents); (**b**) MS2 (changing Mangenese contents); (**c**) MS3 (changing Silicon contents), Figure S4: Composition of phases w.r.t elements (**a**) MC_ETA; (**b**) M3C2;(**c**) M7C3; (**d**)Cementite; (**e**) M23C6; (**f**) G-phase, Figure S5: Site fractions of phases w.r.t elements (**a**) MC_ETA, (**b**) M3C2, (**c**) M7C3, (**d**)Cementite, (**e**) M23C6, (**f**) G-phase, Figure S6: (**a**) Volume fraction Austenite and pearlite regarding Carbon; (**b**) Volume fraction Austenite and pearlite regarding Mn; (**c**) Volume fraction Austenite and pearlite regarding Silicon, Figure S7: Stress-strain calculation for SA508 (**a**); Silicon, (**b**); Manganese; (**c**) Carbon, Figure S8: Scheil solidification diagram for the SUS304 composition and their effect on solidifications.
