# Supplementary material for: Strategies Regarding High-Temperature Applications w.r.t Strength, Toughness, and Fatigue Life for SA508 Alloy
Source: Materials (Basel). 2021 Apr 13;14(8):1953. doi: 10.3390/ma14081953 (PMC8069931; doi:10.3390/ma14081953)
Supplement: Supplementary file 1 [file materials-14-01953-s001.pdf]

materials

Supplementary material

Strategies Regarding High-Temperature Applications w.r.t Strength, Toughness, and Fatigue Life for SA508 Alloy

Muhammad Raies Abdullah [1], Cai Hongneng [1], and Fang Liang [1,2,*]

[1] Key Laboratory for Mechanical Behavior of Materials, Xi'an Jiaotong University, Xi'an 710049, China; hntsai@mail.xjtu.edu.cn (C.H.); raieskhan@stu.xjtu.edu.cn (M.R.A.)
[2] School of Mechanical & Electrical Engineering, Xiamen University Tan Kah Kee College, Zhangzhou 363105, China
* Correspondence: fangl@xjtu.edu.cn; Tel.: +(86)-29-8266-5479

1. Simulation Conditions for Optimization

We use Jmat-Pro (version 7) and Thermo-Calc software (2019a) for simulations, so we some assumptions for these calculations. Thermocalc is working on Gibbs's free energy models [1] for equilibrium conditions. For mechanical calculations related to SA508 analysis, it was assumed at γ-FCC, grain size is 10–15 μm, and calculations were performed at 650 K temperature. For stress-strain calculations, the strain rate was assumed to be 0.1 at 650 K temperature.

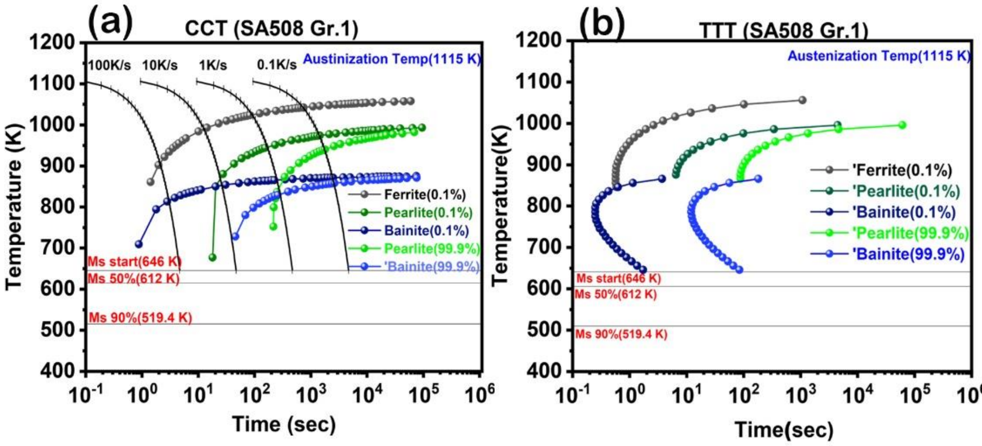

Figure S1. Jmat Pro calculation for SA508 grade steel for RPV (**a**) Continuous Cooling Transformation (CCT) for SA508 (**b**) Isothermal transformation (TTT) for SA508 steel.

Creep is reflected to be a temperature-dependent process and satisfies the power-law equation [2].

$$\varepsilon = A(\frac{\sigma}{E})\exp(\frac{-Q}{RT}) \quad (S1)$$

T is the temperature, σ is significant stress, E is the Modulus, and A is a material-dependent constant; however, R, Q, and n are gas constant, activation energy, and stress exponent. R, T, and A are constants or known, so only Q, n, E, and σ are responsible parameters that change the material's steady-state creep rate.

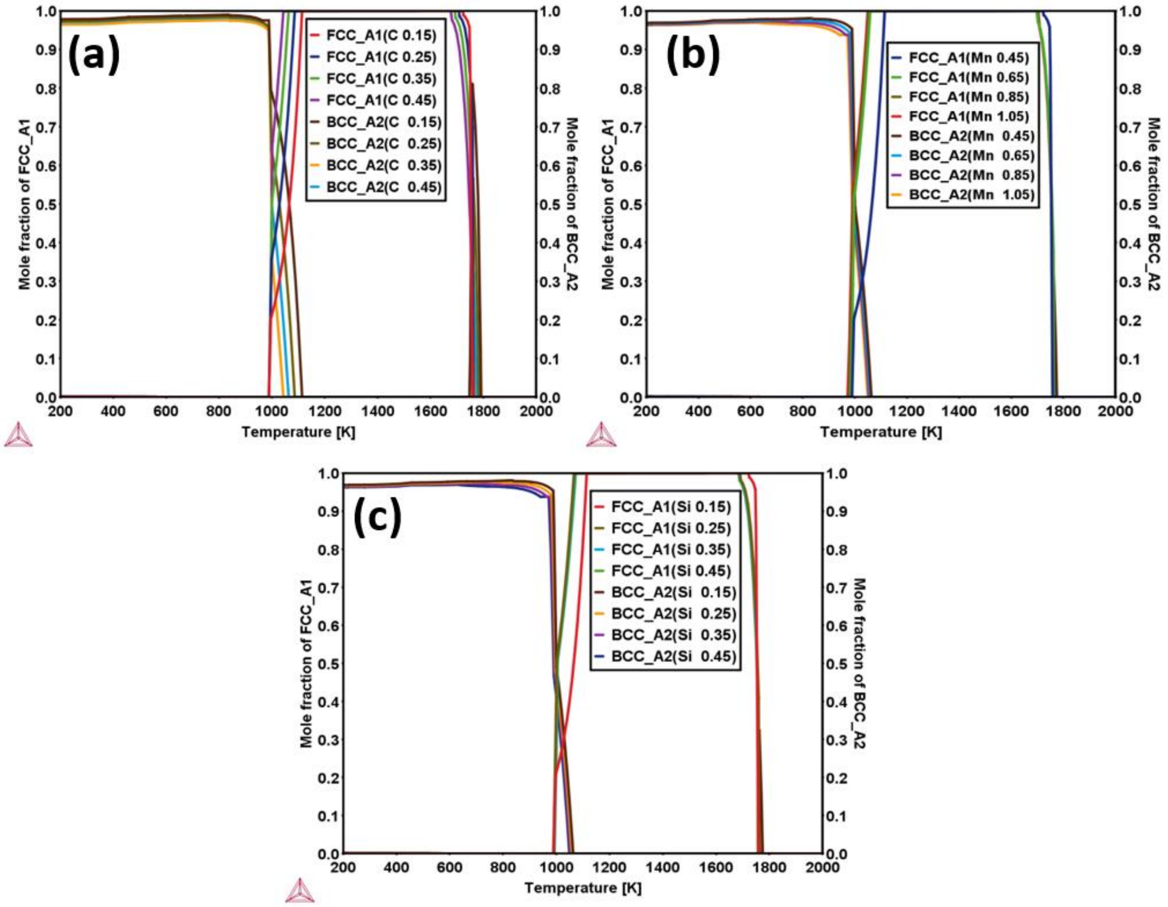

Figure S2. Ac_3(ferrite to Austenite transition temperature) and Ac_4(Austinite to Ferrite transition temperature) temperature calculations (**a**) FCC and BCC for MS1 samples; (**b**) FCC and BCC for MS2 samples; (**c**) FCC and BCC for MS3 samples.

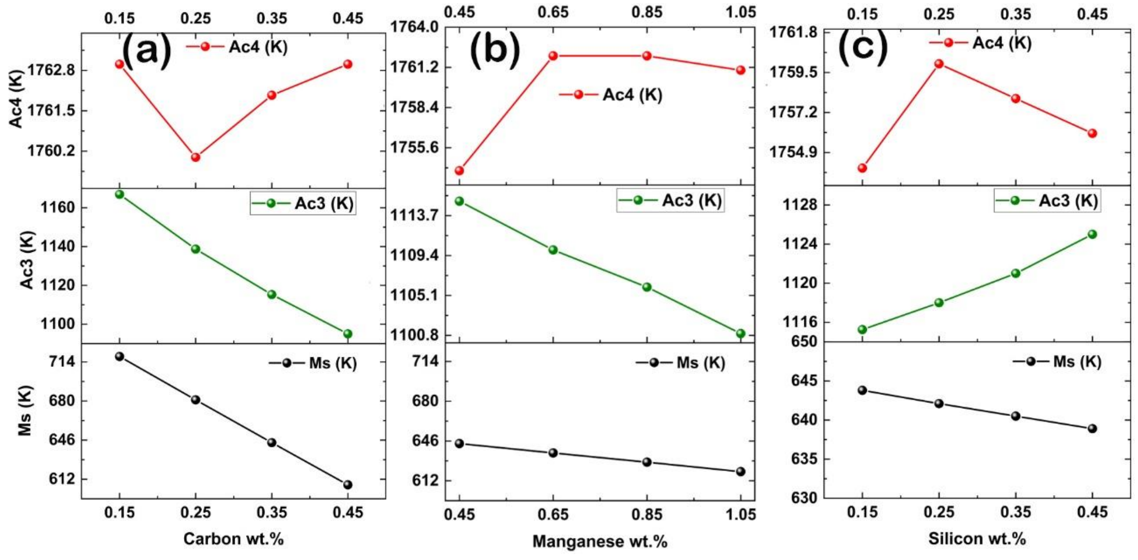

Figure S3. Martensite temperature start (Ms), Ac_3 and Ac_4 temperatures w.r.t (**a**) MS1 (changing Carbon contents); (**b**) MS2 (changing Mangenese contents); (**c**) MS3 (changing Silicon contents).

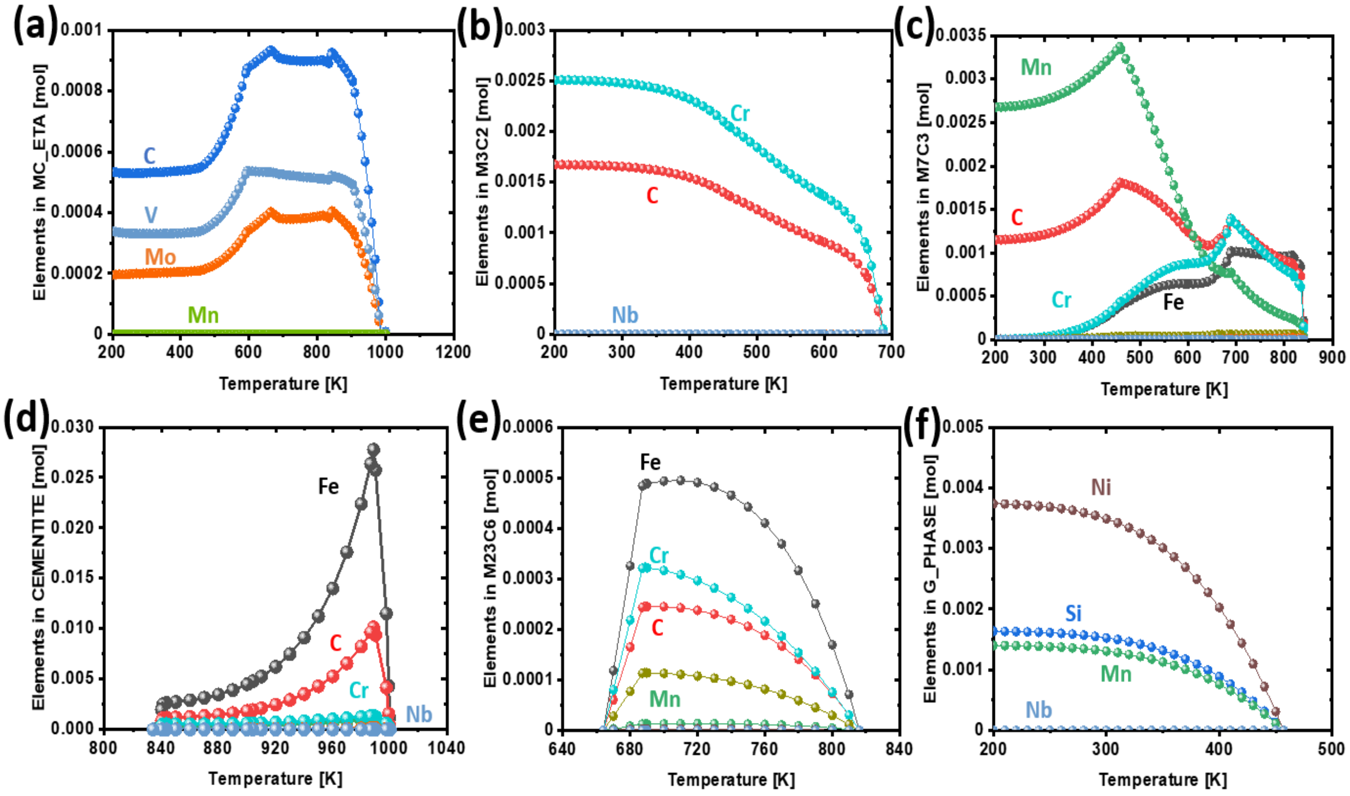

Figure S4. Composition of phases w.r.t elements (**a**) MC_ETA; (**b**) M3C2;(**c**) M7C3; (**d**)Cementite; (**e**) M23C6; (**f**) G-phase.

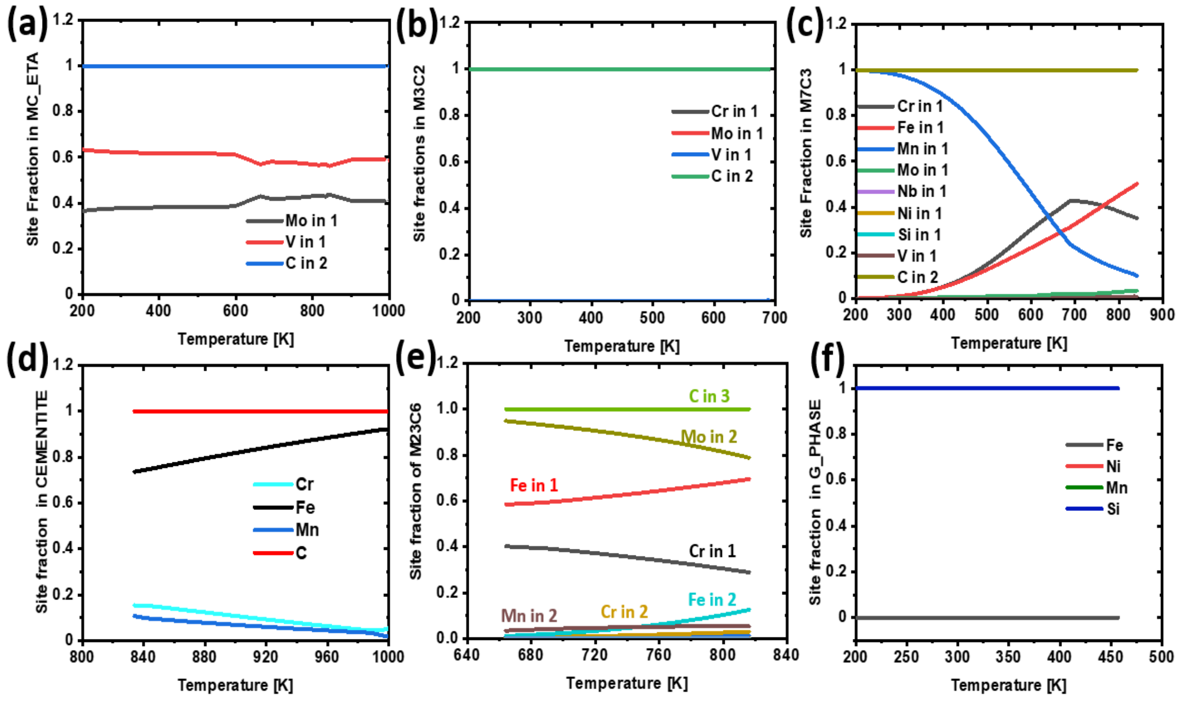

Figure S5. Site fractions of phases w.r.t elements (**a**) MC_ETA, (**b**) M3C2, (**c**) M7C3, (**d**)Cementite, (**e**) M23C6, (**f**) G-phase.

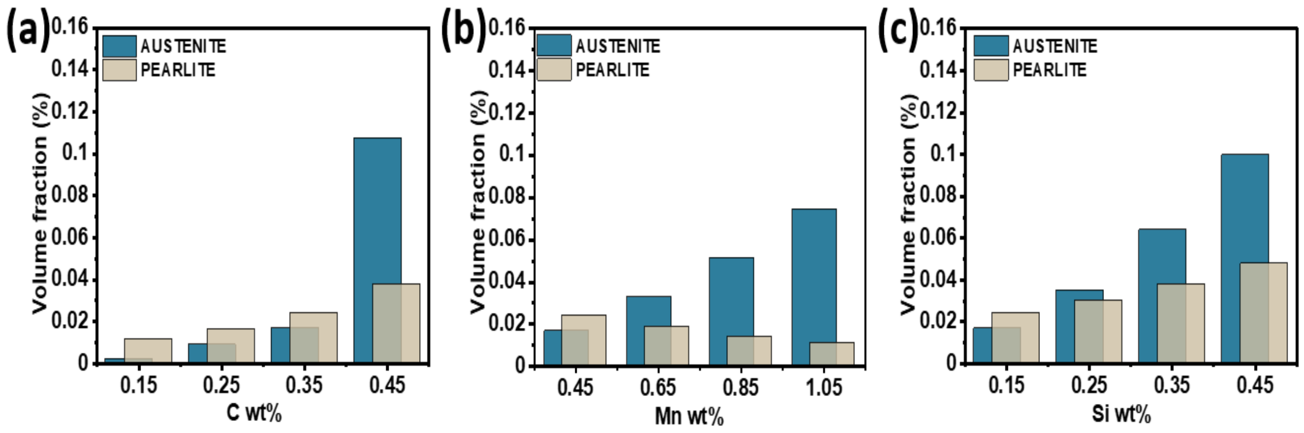

Figure S6. (**a**) Volume fraction Austenite and pearlite regarding Carbon; (**b**) Volume fraction Austenite and pearlite regarding Mn; (**c**) Volume fraction Austenite and pearlite regarding Silicon.

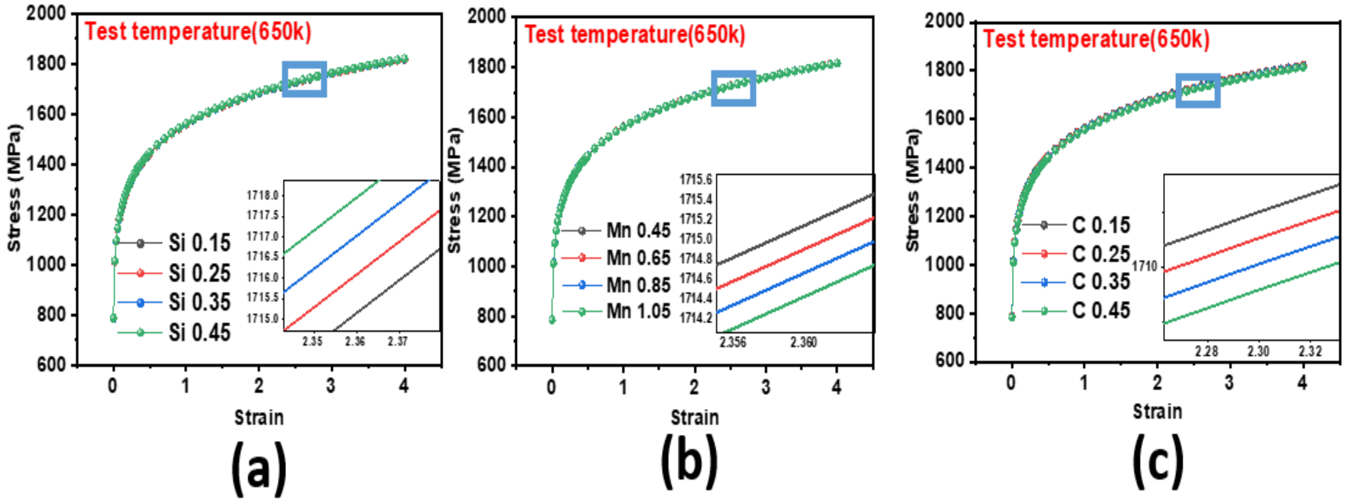

Figure S7. Stress-strain calculation for SA508 (**a**); Silicon, (**b**); Manganese; (**c**) Carbon.

2. Fatigue Equations

Fatigue is an effect of permanent microplastic deformation when a material is exposed to cyclic loading. This often occurs together with thermal and/or corrosive attack owing to the presence of oxygen and hydrogen, causing a phenomenon known as stress corrosion cracking [3].

$$\frac{\Delta\epsilon}{2} = \frac{\Delta\epsilon_e}{2} + \frac{\Delta\epsilon_p}{2} = \frac{\sigma_f'}{E}(2N)^b + \epsilon_f'(2N)^c \quad \text{(S2)}$$

In this equation $\Delta\epsilon$, σ_f' ,ϵ_f', E, and $2N$ are strain amplitude, fracture stress, fracture strain, Young`s Modulus and number of cycles respectively, where b(−0.1) and c(−0.9) are user defined constants [4].

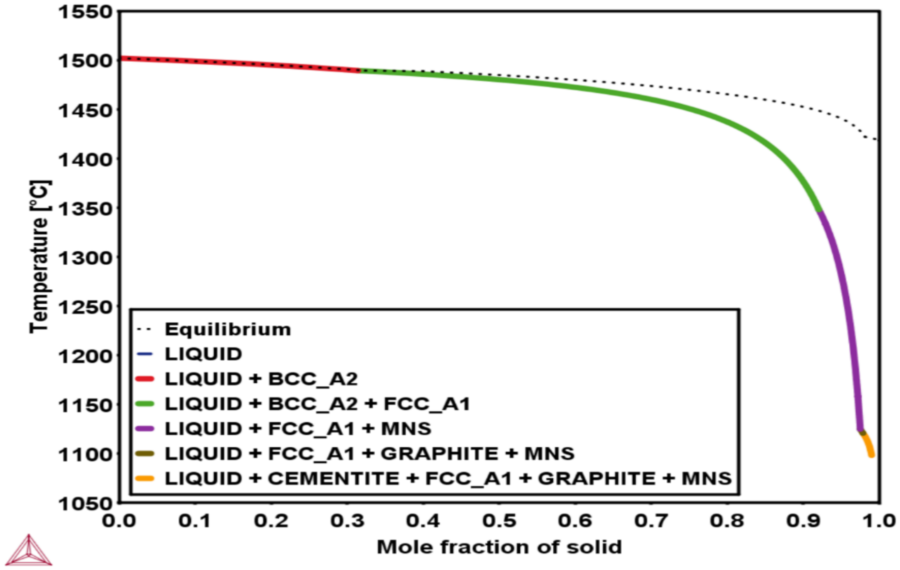

Figure S8. Scheil solidification diagram for the SUS304 composition and their effect on solidifications.

References

1. Chen, H.L.; Mao, H.; Chen, Q. Database development and Calphad calculations for high entropy alloys: Challenges, strategies, and tips. *Mater. Chem. Phys*. **2018**, *210*, 279–290. https://doi.org/10.1016/j.matchemphys.2017.07.082.
2. Miodownik, A.P.; Li, X.; Saunders, N.; Schille, J.-P. Modelling of Creep in Nickel Based Superalloys, 6th Int. Charles Parsons Turbine Conf. (2003). Available online: http://www.sentesoftware.co.uk/media/2555/creep.pdf.
3. Raabe, D.; Tasan, C.C.; Olivetti, E.A.Strategies for improving the sustainability of structural metals. *Nature* **2019,** *575*, 64–74. https://doi.org/10.1038/s41586-019-1702-5.
4. Guo, Z.; Saunders, N.; Miodownik, P.; Schillé, J.P. Modeling material properties of lead-free solder alloys. *J. Electron. Mater.* **2008**, *37*, 23–31. https://doi.org/10.1007/s11664-007-0218-1.
